# Required Gene Set for Autotrophic Growth of *Clostridium autoethanogenum*

**DOI:** 10.1128/aem.02479-21

**Published:** 2022-03-14

**Authors:** Craig Woods, Christopher M. Humphreys, Claudio Tomi-Andrino, Anne M. Henstra, Michael Köpke, Sean D. Simpson, Klaus Winzer, Nigel P. Minton

**Affiliations:** a Clostridia Research Group, BBSRC/EPSRC Synthetic Biology Research Centre (SBRC), Biodiscovery Institute, School of Life Sciences, The University of Nottingham, Nottingham, United Kingdom; b Centre for Analytical Bioscience, Advanced Materials and Healthcare Technologies Division, School of Pharmacy, The University of Nottingham, Nottingham, United Kingdom; c BBSRC/EPSRC Synthetic Biology Research Centre (SBRC), School of Mathematical Sciences, The University of Nottingham, Nottingham, United Kingdom; d LanzaTech, Inc., Skokie, Illinois, USA; e NIHR Nottingham Biomedical Research Centre, Nottingham University Hospitals NHS Trust and the University of Nottingham, Nottingham, United Kingdom; University of Tokyo

**Keywords:** Tn-Seq, TraDIS, acetogen, chemoautotrophy, *Clostridium autoethanogenum*, gene essentiality, minimal genome, transposon sequencing, transposons, Wood-Ljungdahl

## Abstract

The majority of the genes present in bacterial genomes remain poorly characterized, with up to one-third of those that are protein encoding having no definitive function. Transposon insertion sequencing represents a high-throughput technique that can help rectify this deficiency. The technology, however, can only be realistically applied to those species in which high rates of DNA transfer can be achieved. Here, we have developed a number of approaches that overcome this barrier in the autotrophic species Clostridium autoethanogenum by using a *mariner*-based transposon system. The inherent instability of such systems in the Escherichia coli conjugation donor due to transposition events was counteracted through the incorporation of a conditionally lethal *codA* marker on the plasmid backbone. Relatively low frequencies of transformation of the plasmid into *C. autoethanogenum* were circumvented through the use of a plasmid that is conditional for replication coupled with the routine implementation of an Illumina library preparation protocol that eliminates plasmid-based reads. A transposon library was then used to determine the essential genes needed for growth using carbon monoxide as the sole carbon and energy source.

**IMPORTANCE** Although microbial genome sequences are relatively easily determined, assigning gene function remains a bottleneck. Consequently, relatively few genes are well characterized, leaving the function of many as either hypothetical or entirely unknown. High-throughput transposon sequencing can help remedy this deficiency, but is generally only applicable to microbes with efficient DNA transfer procedures. These exclude many microorganisms of importance to humankind either as agents of disease or as industrial process organisms. Here, we developed approaches to facilitate transposon insertion sequencing in the acetogen Clostridium autoethanogenum, a chassis being exploited to convert single-carbon waste gases CO and CO_2_ into chemicals and fuels at an industrial scale. This allowed the determination of gene essentiality under heterotrophic and autotrophic growth, providing insights into the utilization of CO as a sole carbon and energy source. The strategies implemented are translatable and will allow others to apply transposon insertion sequencing to other microbes where DNA transfer has until now represented a barrier to progress.

## INTRODUCTION

Although microbial genome sequences are relatively easily determined, assigning gene function remains a bottleneck. Consequently, relatively few genes are well characterized, leaving the function of many as either hypothetical or entirely unknown. Thus, even the Syn 3.0 minimal genome retains 149 genes (32%) of unknown function (1). A greater understanding of gene functionality can be gleaned through the deployment of high-throughput transposon sequencing. This technique is characterized by the simultaneous Illumina sequencing of the site of transposon insertion in pooled mutant libraries by using a sequencing primer specific to the transposon-chromosome junction. If the library consists of a sufficiently high number of unique insertions, then the required gene set for the growth conditions used can be inferred since unrepresented or underrepresented genes are likely to be essential. There are several names for this type of approach, including the first four, all published in 2009: TraDIS (2), HITS (3), Tn-Seq (4), and INSeq (5). All of these techniques aim to identify the position and quantity of transposon mutants and are collectively referred to as transposon insertion sequencing (TIS) (6).

The deployment of TIS typically is largely dependent on high-frequency DNA transfer. This excludes its application to many microbial species. Anaerobic bacteria, and in particular members of the genus *Clostridium*, are of both medical and industrial importance but generally display low rates of DNA transfer. This has limited the exploitation of TIS in this grouping, where to date TIS has only been applied (7) to the pathogen Clostridioides difficile (formerly Clostridium difficile). One group of bacteria with increasing importance are the anaerobic acetogens, typified by Clostridium autoethanogenum. Acetogens possess the Wood-Ljungdahl pathway (WLP), or reductive acetyl coenzyme A (acetyl-CoA) pathway, which allows the fixation of CO and CO_2_ (8). Suggested to be the earliest autotrophic pathway (9), it is the most energy efficient of the seven known carbon fixation pathways since it conserves energy, while all others require its input (10). Reducing equivalents needed for metabolic processes are obtained either from H_2_ or CO using hydrogenases or CO dehydrogenase (CODH), respectively. Carbon is fixed via the Eastern branch of the pathway, where through a series of cobalamin- and tetrahydrofolate-dependent reactions, CO_2_ is reduced to a methyl group. The methyl group from the Eastern branch is then combined with CO to form acetyl-CoA, which is the root of subsequent anabolic reactions (11–14).

While the majority of acetogens synthesize acetate as the sole fermentation product, some, typified by *C. autoethanogenum*, naturally produce industrially relevant compounds such as 2,3-butanediol and ethanol, the latter on a commercial scale (15). Commercial efforts to extend the product range further are ongoing, with isopropanol being a notable example (16). *C. autoethanogenum* is one of the best understood autotrophic acetogens with a manually annotated genome (17, 18) and has been subjected to transcriptomic and proteomic analyses (19).

In the current study, we sought to maximize the benefit of available *C. autoethanogenum* genome data through implementation of TIS. However, as DNA transfer into *C. autoethanogenum* is only possible at relatively low frequencies, a number of essential modifications to the procedure were required. Specifically, the use of a conditional replicon and an inducible orthogonal expression system to control production of transposase allows the controlled generation of a large mutant library from a small number of initial transconjugant colonies. Additionally, the incorporation of I-SceI recognition sequences into the delivery vehicle provided a mechanism to eliminate those mini-transposon sequences still present on autonomous copies of the plasmid during the transposon mutant library preparation stage. These adaptations have allowed a thorough genetic analysis of the WLP in *C. autoethanogenum* and, for the first time, the determination of the essential gene set required for growth on CO as a sole carbon and energy source.

## RESULTS AND DISCUSSION

### Control of transposition.

A fundamental requirement of an effective transposon delivery system is that transposition should preferentially take place in the target strain and not in the donor strain. A previously described clostridial system exploited the Clostridioides difficile alternate sigma factor TcdR (20) and one of the only two promoters it recognizes, the P*_tcdB_* promoter of the toxin B gene (*tcdB*). By generating a derivative of Clostridium acetobutylicum in which the TcdR-encoding *tcdR* gene was inserted into the genome at the *pyrE* locus, any subsequently introduced gene that was placed under the control of the P*_tcdB_* promoter is expressed. We postulated that this system would be ideal for tightly regulating the transposase.

**FIG 1:**
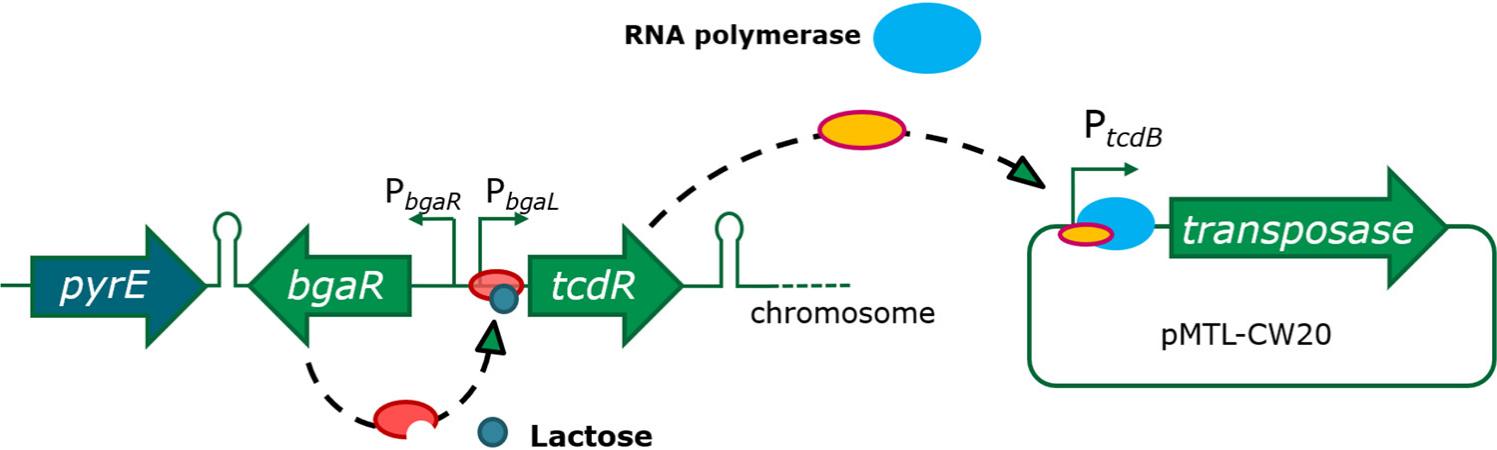
TcdR-mediated orthogonal expression. In *C. autoethanogenum* C24, *tcdR* is under the control of the lactose-inducible promoter system *bgaR*-P*_bgaL_* from C. perfringens. In this way, the P*_tcdB_* promoter can be induced indirectly via the inducible expression of *tcdR* from the chromosome.

For exploitation in *C. autoethanogenum*, further control was engineered into the system by placing expression of *tcdR* (Fig. 1) under the control of a lactose-inducible promoter, P*_bgaL_*, previously shown to be functional in the closely related species Clostridium ljungdahlii (21). Accordingly, the P*_bgaL_* promoter, together with the necessary *bgaR* gene, which encodes a transcriptional regulator, was positioned 5′ to the *tcdR* gene, and the DNA module created (*bgaR*-P*_bgaL_*::*tcdR*) was integrated into the *C. autoethanogenum* chromosome at the *pyrE* locus by allele-coupled exchange (ACE) (21–24). This involved restoring a uracil-requiring Δ*pyrE* mutant strain to prototrophy concomitant with genomic insertion of *bgaR*-P*_gaL_*::*tcdR* using the ACE plasmid pMTL-CH20lactcdR. Successful mutant generation was confirmed by PCR analysis and Sanger sequencing of the amplified DNA, and the resulting strain was termed *C. autoethanogenum* C24.

**FIG 2:**
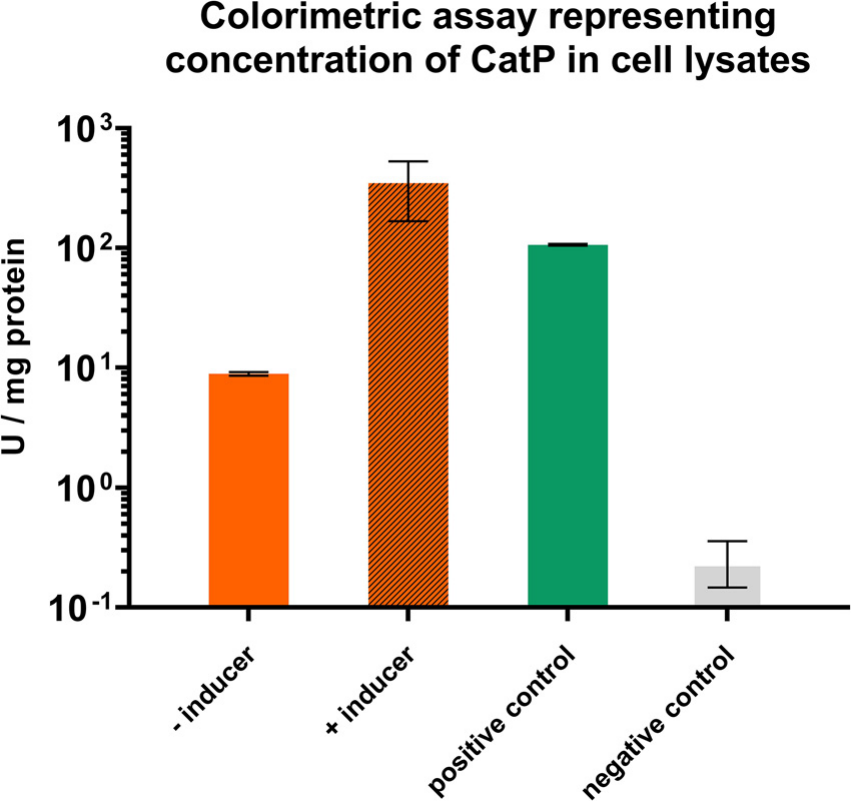
Chloramphenicol acetyltransferase (CAT) assay of lactose-inducible orthogonal system. Expression from P*_tcdB_* was quantified using a CAT assay. Three plasmids were conjugated into *C. autoethanogenum* P*_bgaL__tcdR* (*C. autoethanogenum* C24), with each plasmid harboring *catP* under the control of either P*_tcdB_*, P*_thl_* (positive control), or no promoter (negative control). The strain harboring the P*_tcdB_* plasmid was tested with and without the addition of 10 mM lactose, while the remaining plasmids were tested without lactose. The data shown are the result of biological triplicates, with error bars showing the standard deviation.

**FIG 3:**
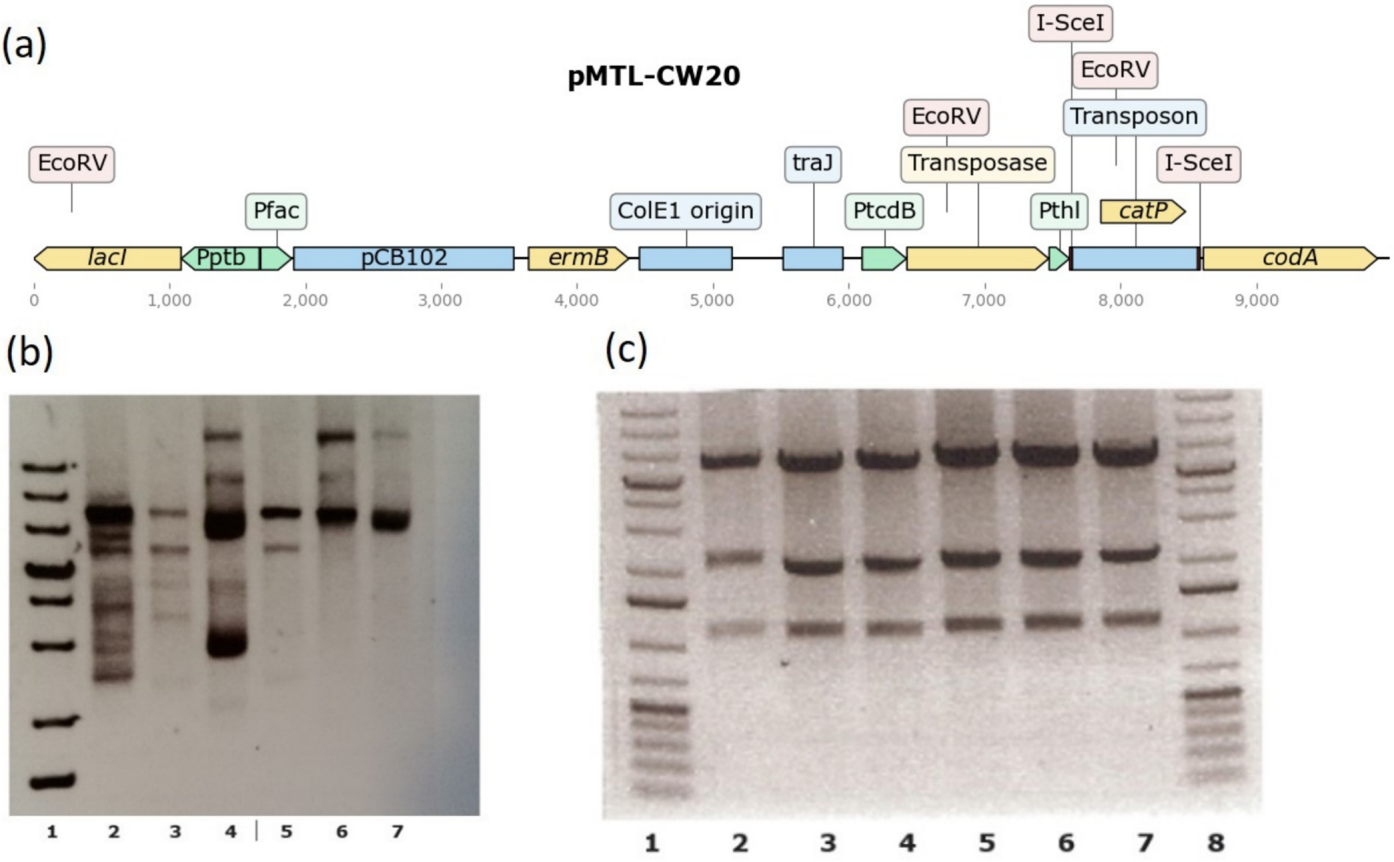
Transposon delivery plasmid pMTL-CW20. (a) pMTL-CW20 is based on the pMTL-YZ14 plasmid described in reference 25, using components from the plasmid modular transfer series outlined in reference 50, as well as the *codA* from E. coli and I-SceI recognition sites. Replication occurs in E. coli via the pUC ColE1 origin of replication, and the plasmid can be transferred to clostridial recipients using the *oriT* from RK2 (51). In clostridial hosts, the plasmid is conditionally replicative, where the presence of IPTG is the nonpermissive condition. Transposition is achieved via a hyperactive Himar1 variant (52) which mobilizes a mini-transposon containing the *catP* gene, which confers chloramphenicol and thiamphenicol resistance. A Rho-independent terminator downstream of the *fdx* gene of Clostridium sporogenes resides upstream of *catP*. (b) A verified pMTL-YZ14 plasmid was used to transform E. coli Top10, and transformant colonies were used to inoculate overnight cultures. Plasmids prepared from overnight cultures were extracted and treated with SbfI. Movement of the transposon into various other parts of the vector was found to have occurred (lanes 2, 4, 6, and 7), while only lanes 3 and 5 exhibited the expected band pattern. A Thermo Fisher 1-kb+ ladder is in lane 1. (c) An analogous procedure using EcoRV was later followed using pMTL-CW20 instead of pMTL-YZ14. In this case, all six plasmids exhibited the expected band pattern (lanes 2 to 7), with the Thermo Fisher 1-kb+ ladder shown in lanes 1 and 8.

To confirm that TcdR production could be controlled by the addition of exogenous lactose in strain C24, the Clostridium perfringens *catP* reporter gene encoding a chloramphenicol acetyltransferase (CAT) was cloned downstream of the P*_tcdB_* promoter on an appropriate clostridial shuttle vector. Regulation of the reporter gene was shown to be dependent on the addition of the lactose inducer (Fig. 2). We therefore chose to use this expression system to create our transposon library by placing the transposase under the control of P*_tcdB_* on the transposon delivery plasmid pMTL-CW20 (Fig. 3).

A second feature of pMTL-CW20 designed to control unwanted transposition was based on the provision of a promoterless copy of the Escherichia coli *codA* gene encoding cytosine deaminase to prevent premature transposition in the donor strain. Use of the previously described transposon delivery vector pMTL-YZ14 (25) was characterized by inconsistent frequencies of transfer to the clostridial recipient and/or to variation in the effectiveness with which transposon mutants were generated once in *C. autoethanogenum*. These inconsistencies appeared to correlate with spontaneous plasmid rearrangements in the donor, as evidenced by unexpected DNA fragment profiles on agarose gels of diagnostic digests of the isolated plasmid DNA (Fig. 3). This was assumed to be due to transposition of the mini-transposon from pMTL-YZ14, while in E. coli this represented either insertion into the genome or, as transposition into closed circular autonomous plasmids is preferred, into alternative positions in the vector backbone. The cut-and-paste nature of the transposition event would mean that plasmids would be generated that either no longer carried a mini-transposon or which had been affected in their maintenance or ability to transfer. Similar instabilities have been noted elsewhere (26).

Cytosine deaminase catalyzes conversion of 5-fluorocytosine (5-FC) to the toxic product 5-fluorouracil (5-FU), which ultimately blocks DNA and protein synthesis. On the plasmid pMTL-CW20, *codA* is separated from its P*_thl_* promoter (derived from the thiolase gene of Clostridium acetobutylicum) by the *catP* mini-transposon. Excision of the mini-transposon as a consequence of its transposition leads to expression of *codA*, a lethal event in the presence of exogenously supplied 5-FC. The addition of this feature to pMTL-CW20 improved the reproducibility with which the plasmid was transferred to *C. autoethanogenum* and appeared to prevent the occurrence of plasmid rearrangements (Fig. 3).

### Removal of plasmid-based reads.

The use of suicide vectors for transposon delivery is reliant on high frequencies of DNA transfer. Our initial attempts in *C. autoethanogenum* using a delivery vehicle lacking a Gram-positive replicon yielded just 5 transposon mutants from 3 independent conjugations. To overcome this low frequency of mutant generation, a conditional replicon was utilized, which has been described previously (25). To further remove any residual plasmid from the sequencing library, I-SceI recognition sites were incorporated into pMTL-CW20, which provided a mechanism for removal of plasmid reads at the sequencing library stage. After adapter ligation, an I-SceI restriction is used to cleave the site between the adapter and the library primer binding site on the transposon, making those fragments originating from plasmid DNA unsuitable templates for the subsequent PCR amplification step, as described in a similar strategy (27). Since there is no I-SceI recognition site in the *C. autoethanogenum* genome, transposon insertion sites in the genome will be identified as usual. In the initial transposon library grown on rich medium, 0.2% of reads mapped to the transposon delivery plasmid. This compares favorably with a study on Clostridiodes difficile that used a replicative vector, in which 48% of the reads in the initial rich medium library mapped to the delivery plasmid (7).

### Generation of transposon library and growth under autotrophic conditions.

Approximately 1.3 million colonies were pooled from 125 transposon selection plates and inoculated into 200 mL of rich medium (YTF) supplemented with thiamphenicol and IPTG (isopropyl-β-d-thiogalactopyranoside). After 24 h of growth, genomic DNA (gDNA) was extracted from this culture, and −80°C freezer stocks were made. This first genomic DNA extraction was used to determine the required gene set for growth on rich medium; a total of 100,065 unique insertion sites were found from this sample. The genome length divided by the number of unique insertions is 43.49. Subsequently the freezer stocks were used to restore the mutant pool into a defined medium (PETC) supplemented with pyruvate as the carbon source. The PETC culture was used to inoculate a 1.5-L bioreactor containing fermentation medium that lacked a carbon source. The sole carbon and energy source after the inoculation was provided by CO gas sparged into the bioreactor with a gradual increase of CO. The pyruvate was quickly used up, as shown by high-performance liquid chromatography (HPLC) data (see Table S3 in the supplemental material), and *C. autoethanogenum* instead relied upon fixation of CO. The PETC medium provided no supplementary amino acids and instead relied on the native biosynthesis pathways of *C. autoethanogenum*. Vitamin requirements were met via the addition of Wolfe’s vitamin solution.

Samples for HPLC analysis of metabolites and for possible genomic DNA extraction and TIS analysis were taken on a daily basis. Samples from 72, 144, 168, 336, and 360 h of growth were used for sequencing. These sequencing data were used to determine the required gene set for growth using CO in a defined medium. An insertion index value of lower than 0.0013 was the cutoff for essentiality. Ultimately, the samples from 336 h and 360 h were used to determine the gene set required for growth on CO; these represent the endpoint of the reactor fed batch culture. The reactor endpoint was sequenced, revealing 66,524 unique insertion sites.

### Functions of essential genes under heterotrophic compared with autotrophic conditions.

The functions of candidate essential genes for growth in rich medium and minimal medium with CO as the carbon and energy source were compared using the KEGG database as summarized in Table 1. There were 439 genes (11%) identified as candidate essential genes out of a total of 4,059 genes in the genome for heterotrophic growth on the rich medium YTF, where fructose and yeast extract serve as a carbon and energy source (see Table S1 in the supplemental material). This is comparable to the number of genes in the Syn3.0 genome and close to the 404 reported in Clostridiodes difficile (1, 7). As expected, genes involved in fundamental biological processes such as transcription, translation, DNA replication, and cell division are common in the rich medium essential gene list. Eighteen of the 20 common amino acids have clearly annotated tRNA synthetases that appear essential, except for tyrosine and asparagine. Tyrosine appears to exhibit redundancy via CLAU_1290 (*tyrZ*) and CLAU_1635. There is only one annotated asparagine tRNA synthetase (*asnB*), but it seems likely that there is another present (CLAU_2687) and that together they provide functional redundancy, meaning that both genes are found to be nonessential. CLAU_2687 is currently annotated as a tRNA synthetase class II, but is most likely to be an asparagine-specific tRNA synthetase. Another explanation for the nonessential status of the asparagine tRNA synthetase could be that *C. autoethanogenum* uses a mechanism common to many bacterial and archaeal taxa which entirely lack an asparagine tRNA synthetase. These taxa rely on a nondiscriminating aspartic acid tRNA synthetase followed by an amidotransferase to generate asparagine tRNAs (28).

**TABLE 1:** Functions of *C. autoethanogenum* essential genes

Function	No. of essential genes on^*a*^:	
	Rich medium	Minimal medium + CO
Metabolic pathways	98	156
Ribosome	38	42
Microbial metabolism in diverse environments	33	41
Carbon metabolism	25	30
Aminoacyl-tRNA biosynthesis	21	23
Biosynthesis of amino acids	18	36
Hypothetical	13	24
Peptidoglycan biosynthesis	11	12
Pyruvate metabolism	9	11
Carbon fixation pathways in prokaryotes	9	13
DNA replication	9	11
Amino sugar and nucleotide sugar metabolism	9	9
Oxidative phosphorylation	7	10
Fatty acid metabolism	6	7
Fatty acid biosynthesis	6	7
Propanoate metabolism	6	7
Quorum sensing	5	7
Protein export	5	9
Purine metabolism	5	14
Bacterial secretion system	5	9
RNA degradation	5	7
RNA polymerase	4	4
Pyrimidine metabolism	4	10
Citrate cycle (tricarboxylic acid cycle)	3	7
Phosphotransferase system (PTS)	2	2
Folate biosynthesis	2	5
Biotin metabolism	1	2
Flagellar assembly	1	1
Bacterial chemotaxis	1	2

a Shown are the number of *C. autoethanogenum* essential genes for various KEGG functional categories on the rich medium YTF and the minimum fermentation medium with CO as a carbon and energy source.

The candidate essential gene list for rich medium calls into question several of the annotations in the *C. autoethanogenum* genome. For instance, CLAU_0265 which is annotated as a small acid-soluble spore protein, is required on rich medium, despite that fact that sporulation should not have been required in the library preparation process. The gene must, therefore, have an additional or alternative role. Much functional genomics work has yet to be performed on *C. autoethanogenum* since there are 44 rich medium essential genes annotated as hypothetical proteins (Table S1).

In total, 758 genes (19% of the genome) were predicted to be required for autotrophic growth by the endpoint of the CO-fed reactor (Table S1). This includes all of the “core” gene set, which were also required on rich medium, and all of the genes required to grow on minimal medium lacking amino acids. The core gene set was predicted to be comprised of 439 genes. This means that 319 genes are likely to be required for the synthesis of all amino acids and utilization of CO as a carbon and energy source. As vitamins were provided, their biosynthetic pathways were not expected to be represented; similarly, nitrogen and sulfur were supplied in the medium as ammonium chloride and sodium sulfide, respectively. All genes and their predicted essentiality status in each experimental condition are presented in Table S1. Comparing two predicted required gene lists from different times in an experiment is an imperfect method of deducing condition-specific genes, but the data are nevertheless extremely informative.

### Essential genes of the WLP.

**FIG 4:**
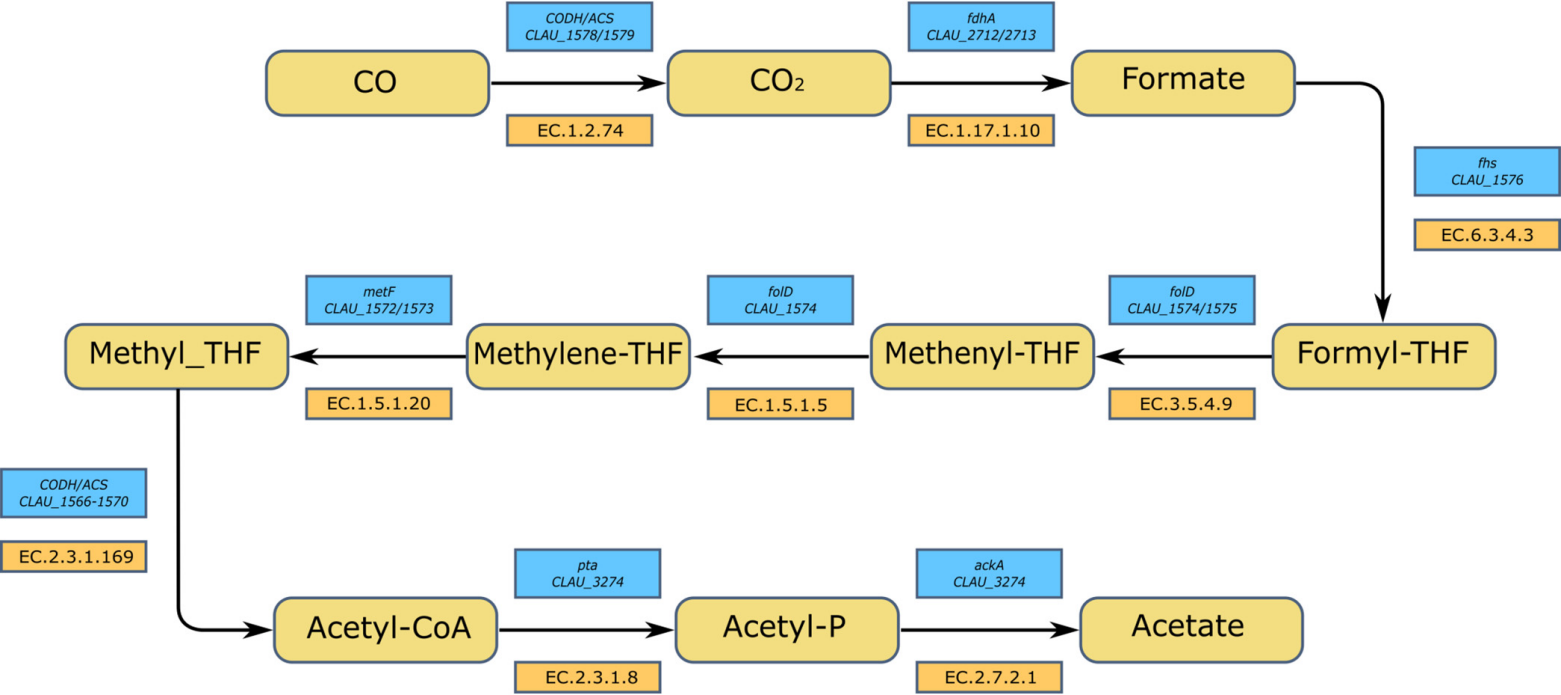
Essential genes of the Wood-Ljungdahl pathway. The route from CO to acetate showing the expected gene/locus tag for each step is illustrated. Each of the locus tags listed was required for growth on CO.

In order to grow using CO as a sole carbon and energy source, it is necessary for *C. autoethanogenum* to use two molecules of CO to form one molecule of acetyl-CoA. Acetyl-CoA consists of a methyl group, a carbonyl group, and the CoA cofactor. The methyl group is supplied by the action of the bifunctional CODH enzyme, which oxidizes CO to CO_2_. This CO_2_ molecule then follows the Eastern branch of the WLP before being combined with another CO molecule and CoA by the acetyl-CoA synthase (ACS). It was therefore expected that all the genes involved in the WLP would be required for growth on CO. A complete WLP was indeed found in the list of essential genes and has been mapped out in Fig. 4. The WLP was not required during heterotrophic growth, despite the fact that it is utilized during heterotrophic growth to fix CO_2_ released during glycolysis using the reducing equivalents generated by glycolysis (24).

**FIG 5:**
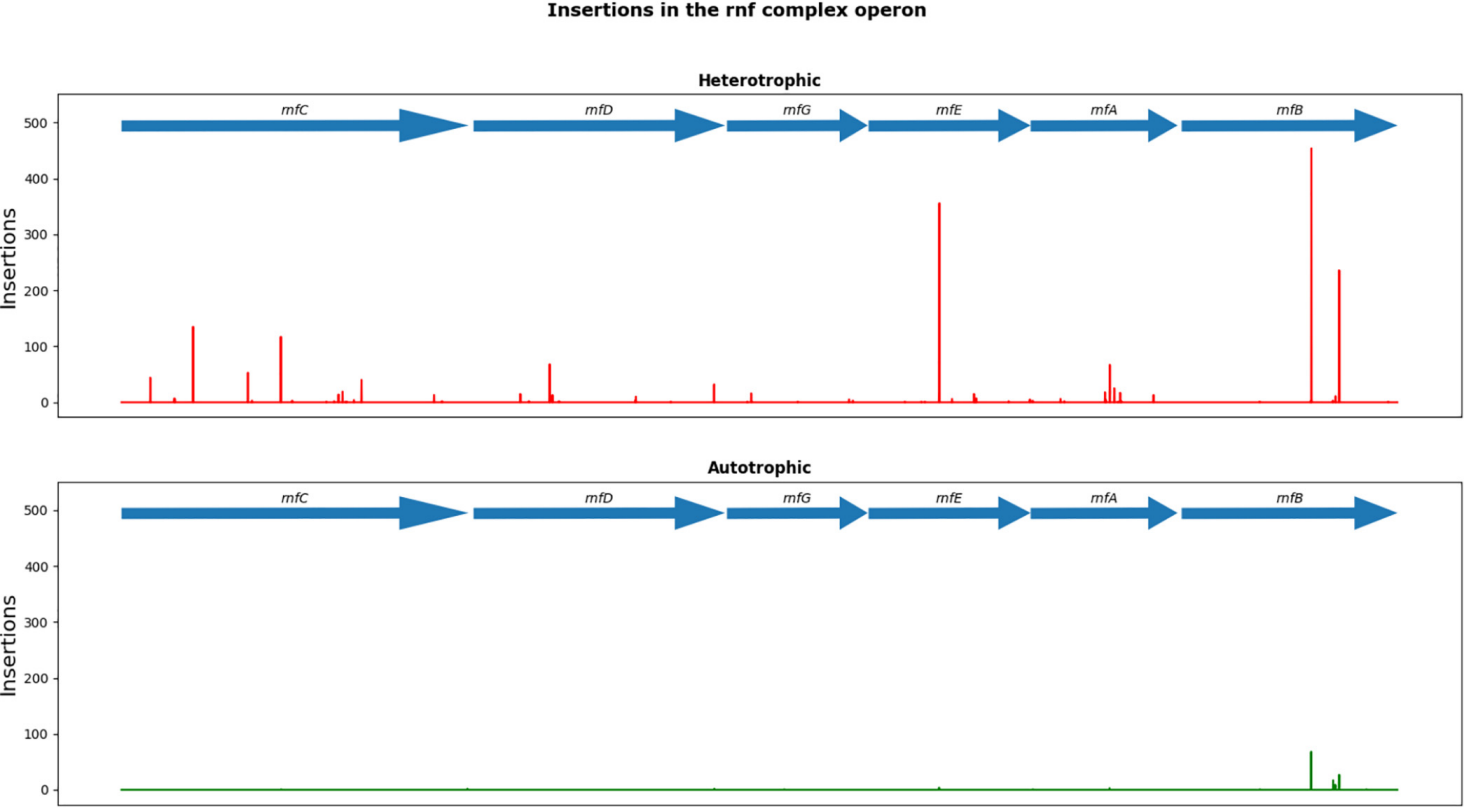
Insertions in the Rnf complex region. Shown is the number of reads detected along the genomic region encoding the Rnf complex under heterotrophic and autotrophic conditions. Insertions are relatively abundant under heterotrophic conditions, implying importance for the complex under autotrophic conditions.

To generate ATP, *C. autoethanogenum* is reliant on generating a transmembrane electrochemical gradient via the intrinsically important (29) Rnf complex and a membrane integral ATP synthase (19, 30). The Rnf complex of *C. autoethanogenum* is encoded by the region CLAU_3144 to CLAU_3149. A comparison of the insertion sites found under heterotrophic and autotrophic conditions for this region is shown in Fig. 5. With exception of *rnfB*, all of the encompassed genes were found to be essential for growth on CO (Fig. 5), confirming previous observations that inactivation of these genes in either *C. ljungdahlii* and Acetobacterium woodii curtailed growth on H_2_ plus CO_2_ (31, 32). Despite *rnfB* being above the insertion index threshold for essentiality on CO, it is significantly underrepresented when the data obtained from cells grown on pyruvate are compared with those for CO (log_2_ fold change = −2.86, *P* = 1.16E−11). It may be the case that *rnfB* encodes a nonessential component of the complex that aids functionality but is not required for it. In methanogens, RnfB has been characterized as an entry point for electrons to the Rnf complex (33).

### The importance of Nfn for autotrophic growth.

In order to further verify the calling of genes essentially under specific conditions using our parameters, a candidate gene was selected for directed CRISPR mutagenesis. The *nfn* gene (CLAU_1539) encodes an electron-bifurcating ferredoxin-dependent transhydrogenase responsible for the production of NADPH from NADH and Fd^2−^, thus recycling NAD^+^. Our TIS data analysis found that the *nfn* gene was nonessential when *C. autoethanogenum* was grown on rich medium or when grown on minimal medium with pyruvate, but when autotrophic conditions were used, the gene was essential. This suggested that a directed CRISPR knockout mutant should be obtainable while the culture is grown under heterotrophic conditions but should fail to survive when transferred to autotrophic conditions. A CRISPR in-frame deletion mutant of *nfn* (Δ*nfn*) was created that was viable on rich medium and on minimal medium with pyruvate as a carbon source, but was unable to grow when CO was the sole carbon and energy source.

Initially the Δ*nfn* strain was characterized in serum bottles, using minimal PETC medium and either 10 mM sodium pyruvate or 10^5^ Pa of CO in the headspace as the carbon and energy source. Serum bottles were inoculated with 1 mL (1:50 inoculum) of a late-exponential culture grown in the anaerobic cabinet on minimal medium with fructose as a carbon source. The cultures grown on pyruvate grew similarly to the wild-type control; however, no evidence of growth was evident when CO was used instead of pyruvate as the carbon and energy source. This inability to utilize CO as a carbon and energy source was further demonstrated on a larger scale using a fed-batch continuous stirred tank reactor (CSTR) experiment, whereby a 1.5-L culture was inoculated with 150 mL of an early-exponential culture grown on minimal medium and pyruvate. The pH was controlled with NaOH and H_2_SO_4_, and sparged through continual addition of nitrogen at a rate of 60 mL/min. At the time of inoculation, 5 mM sodium pyruvate was added to the culture. Once an optical density at 600 nm (OD_600_) of approximately 0.3 had been reached, CO was introduced at a rate of 10 mL/min. In the case of the wild-type culture, the strain was able to adapt to the CO carbon and energy source, and after 48 h, the OD continued to increase after the pyruvate had been depleted. In the case of the *nfn* mutant, the culture was not able to adapt to utilizing CO, and the optical density rapidly declined following depletion of the pyruvate (data not shown).

### Assessment of metabolic modeling-derived gene essentiality.

Experimentally confirmed gene essentiality for growth on minimal medium supplemented with CO was compared against the predicted essentiality calculated from a metabolic model of *C. autoethanogenum* by means of flux balance analysis (FBA) (34, 35) (see Table S2 in the supplemental material). To that end, the confusion matrix was generated (Table 2) to calculate Matthew’s correlation coefficient (MCC), a robust metric ranging from −1 to +1 used to evaluate binary classifications (such as essential or nonessential genes) (36). An MCC of 0.34 was obtained, which compares unfavorably to a similar study in E. coli, where a value of 0.69 was reported (37, 38) (for the well-curated model *i*JO1366 and by using thermodynamics and multiomics constraints). The lower value for *C. autoethanogenum* is a measure of the comparatively limited genome annotation in this organism. However, Table 2 shows that the model does have predictive power, with 87 genes being correctly predicted to be essential.

**TABLE 2:** Confusion matrix for gene essentiality comparison

Predicted genes	Exptl result for genes^*a*^	
	Essential	Nonessential
Essential	TP = 87	FP = 59
Nonessential	FN = 92	TN = 294

a TP, true positive; FP, false positive; FN, false negative; TN, true negative. A perfect correlation (MCC = +1) would require TP = 179, FP = 0, FN = 0, and TN = 353.

### Essentiality in the WLP.

The generation of the methyl group from CO first requires its oxidation to CO_2_ by a CODH. *C. autoethanogenum* possesses three such enzymes in its genome, namely, CLAU_1578/CLAU_1579 (*acsA*), CLAU_2924 (CAETHG_3005) (*cooS1*), and CLAU_3807 (CAETHG_3899) (*cooS2*) (18). Their independent interruption via ClosTron mutagenesis (39) showed that autotrophic growth was only abolished in the *acsA* knockout mutant, suggesting it is the only CODH required for growth on either CO or CO_2_. Our data validate this conclusion, with only *acsA* being predicted as required for growth on CO but not on YTF. The remaining putative CODH genes were required under neither condition, and there was no substantial change in insertion index between YTF and CO conditions. The conditionally essential CODH encoded by *acsA* has an internally translated stop codon (TGA) not found in the equivalent genes of related organisms and can alternatively be thought of as two open reading frames (ORFs) (CLAU_1578 and CLAU_1579), although it appears that CLAU_1579 makes no separate product. It has been demonstrated that *acsA* can be translated either as a 44-kDa protein or as a 69-kDa protein, depending on whether the TGA internal stop codon is the end of translation or whether it causes the incorporation of a selenocysteine residue (24). It appears from our data that both ORFs are required under autotrophic conditions. Thus, the 44-kDa protein alone does not appear to be sufficient for autotrophy, and cells apparently require the 69-kDa protein to be autotrophic.

There are three putative formate dehydrogenases in the *C. autoethanogenum* genome, encoded by CLAU_0081, CLAU_2712/CLAU_2713 (*fdhA*), and CLAU_2907. Of these, *fdhA* alone appears to be essential only on CO, while the remaining two genes are required under neither tested condition. The most important formate dehydrogenase is therefore the one encoded by *fdhA*, which is found in a complex with an NADP-specific electron-bifurcating [FeFe]-hydrogenase (Hyt) (40). Two of the three putative formate dehydrogenases are selenoenzymes, which may be higher efficiency than the cysteine-containing analogues: it is therefore tempting to speculate that the non-selenoenzyme formate dehydrogenase may be present as a backup for low-selenium conditions (41). However, it appears from our data that neither CLAU_0081 nor CLAU_2907 could provide sufficient activity in the *fdhA* mutants for them to not be outcompeted, causing *fdhA* to appear essential under autotrophic conditions.

The steps from formate to methyl-THF are catalyzed by the products of CLAU_1572 to CLAU_1576, which all appear to be required for growth on CO. CLAU_1574 and CLAU_1576 additionally appear to also be required for growth on the rich medium.

The methyl group of methyl-THF is transferred to the corrinoid iron-sulfur protein (CoFeSp) cofactor before being combined with the carbonyl group supplied by another molecule of CO by the action of the ACS (acetyl-CoA synthase). The ACS is encoded by the region CLAU_1566 to -1570, in which CLAU_1566, CLAU_1568, and CLAU_1569 were only required on CO, whereas CLAU_1567 and CLAU_1570 were required on CO and on YTF.

### Essentiality in the metabolism from acetyl-CoA.

There are four main carbon compounds at the endpoints of metabolism for *C. autoethanogenum*: acetate, ethanol, 2,3-butanediol, and lactate. The route to acetate from acetyl-CoA proceeds through acetyl phosphate, catalyzed by the enzymes phosphate acetyltransferase (encoded by *pta*) and acetate kinase (encoded by *ackA*). Both *pta* and *ackA* were found to be essential when growing on rich medium. The step from acetyl phosphate to acetate regenerates ATP, and so this pathway may be required for energy generation.

However, *pta* has been knocked out in *C. ljungdahlii* (42–44), where it significantly impaired growth rates and acetate formation. The *C. ljungdahlii pta* knockout may be viable only because of a second putative phosphate acetyltransferase (WP_063556670.1), which is annotated as a bifunctional enoyl-CoA hydratase/phosphate acetyltransferase and has no homolog in *C. autoethanogenum*. The bifunctional enoyl-CoA hydratase/phosphate acetyltransferase may be producing sufficient ATP for cells to be viable. The absence of an alternative phosphate acetyltransferase in *C. autoethanogenum* is likely the cause of the essentiality of *pta* in our data.

The route to ethanol can proceed from acetyl-CoA either straight to acetaldehyde and then to ethanol or via acetate, then acetaldehyde, and finally ethanol. The more direct route from acetyl-CoA to acetaldehyde is catalyzed by an acetaldehyde dehydrogenase (EC 1.2.1.10), which could be encoded by an estimated five genes within the *C. autoethanogenum* genome (CLAU_1772, CLAU_1783, CLAU_3204, CLAU_3655, CLAU_3656), none of which appears to be required under either growth condition. This could represent redundancy between these genes, which further knockout studies could aim to confirm, or it could be that this route to ethanol is not required. The alternative route to ethanol via acetate is similar in that there are two predicted genes (CLAU_0089 and CLAU_0099) that could encode an aldehyde ferredoxin oxidoreductase (AOR) (EC 1.2.7.5), but neither of them appears to be essential under either growth condition. In this case, the result is best explained by a lack of biological necessity for this reaction since it has been shown that a double-AOR-knockout strain was still viable autotrophically (24).

There are two candidate genes encoding pyruvate synthase enzymes for formation of pyruvate from acetyl-CoA (CLAU_0896 and CLAU_2947), of which only CLAU_2947 appears to be required; this is true under both growth conditions. All of the genes encoding functions for the pathways leading to lactate and 2,3-butanediol appear nonessential. In the case of the conversion of acetolactate to acetoin and in the production of lactate utilizing NADH, there appears to be only one gene encoding each of the relevant functions (CLAU_2851 and CLAU_1108, respectively); in these cases, redundancy is unlikely to be the reason for their nonessential status, meaning it is more likely these are unnecessary biological routes.

The successful application of TIS to *C. autoethanogenum* has provided a wealth of information on gene essentiality in this industrially important acetogen and represents the most thorough analysis of its kind performed to date in clostridia. The essentiality status of all *C. autoethanogenum* genes can now be consulted (in Table S1) before directed knockouts are attempted. Overall, our findings highlight that TIS represents a powerful functional genomics tool that can be applied to less genetically tractable organisms using the methods applied here. The presented data allow a confident determination of the WLP genes of *C. autoethanogenum* and open up future avenues of investigation into the genes that are essential for autotrophic growth with no obvious reason as to why.

## MATERIALS AND METHODS

### Microbiology.

E. coli DH5α (NEB) was used for all for cloning, and sExpress (44) was used as a conjugal donor. Strains were cultured at 37°C in LB broth with appropriate antibiotic supplementation and 5-FC in a shaking incubator or on LB agar in a static incubator. *C. autoethanogenum* was cultured and manipulated in an anaerobic workstation (Don Whitley) with an atmosphere of 80% nitrogen, 10% carbon dioxide, and 10% hydrogen at 37°C. The three media used were YTF (see Tables S4 to S7 in the supplemental material), ATCC medium 1754 (see Tables S8 to S10 in the supplemental material) and fermentation medium (see Tables S11 to S13 in the supplemental material). YTF medium was composed of yeast extract (10 g/L), tryptone (16/L), fructose (10 g/L), NaCl (0.2 g/L), H_3_BO_3_ (100 μg/L), MnCl_2_ · 4H_2_O (230 μg/L), FeCl_2_ · 4H_2_O (78 μg/L), CoCl_2_ · 6H_2_O (103 μg/L), NiCl_2_ · 6H_2_O (602 μg/L), ZnCl_2_ (78 μg/L), CuSO_4_ · 5H_2_O (50 μg/L), AlK(SO_4_)_2_ · 12H_2_O (50 μg/L), Na_2_SeO_3_ (58 μg/L), Na_2_WO_4_ (53 μg/L), Na_2_MbO_4_ · 2H_2_O (52 μg/L), *p*-aminobenzoate (114 μg/L), riboflavin (104 μg/L), thiamine (200 μg/L), nicotinate (206 μg/L), pyridoxin (510 μg/L), pantothenate (104 μg/L), cyanocobalamin (78 μg/L), d-biotin (22 μg/L), folate (48 μg/L), and lipoate (50 μg/L). The fermentation medium contained MgCl_2_ · 6H_2_O (0.5 g/L), CaCl_2_ · 2H_2_O (0.37 g/L), KCl (0.15 g/L), NaCl (0.12 g/L), 85% H_3_P0_4_ (0.38 mL/L), NH_4_Cl (1 g/L), CoCl_2_ (476 ng/L), HBO_4_ (124 ng/L), MnCl_2_ · 4H_2_O (396 ng/L), NaMoO_4_ · 2H_2_O (484 ng/L), Na_2_SeO_3_ (346 ng/L), FeCl_2_ · 4H_2_O (3.87 μg/L), NiCl_2_ · 6H_2_O (238 ng/L), and ZnCl_2_ (138 ng/L).

Plasmids were transferred from sExpress to *C. autoethanogenum* as detailed in reference 45. Briefly, this involved mixing the donor and recipient cultures together and incubating the mixture on a nonselective YTF plate for 20 h at 37°C before harvesting and plating onto selective YTF agar. Antibiotic selection for transposon plasmids was performed using chloramphenicol (25 μg/mL) and erythromycin (500 μg/mL) for E. coli or thiamphenicol (15 μg/mL) and clarithromycin (6 μg/mL) for *C. autoethanogenum*. Kanamycin (50 μg/mL) was used to select for the sExpress donor strain. d-Cycloserine (250 μg/mL) was used to counterselect the sExpress donor strain. 5-Fluorocytosine (5-FC) was supplemented at 30 μg/mL and IPTG at a concentration of 1 mM. Plasmid pMTL-CW20 may be sourced from www.plasmidvectors.com.

### DNA manipulations.

Genomic DNA (gDNA) purifications were performed using bacterial gDNA extraction kits from Sigma-Aldrich. Plasmid DNA was purified with miniprep kits from NEB. Screening PCRs were performed using DreamTaq polymerase (Thermo Fisher). Oligonucleotides were synthesized by Sigma-Aldrich. Sanger sequencing was performed by Source Bioscience.

### Mutant generation using CRISPR.

A CRISPR in-frame deletion vector was designed as previously described using the pMTL40000 CRISPR vector series (46). In this case, we employ the trCas9 nickase variant under the control of the P*_fdx_* promoter from the *C. sporogenes* ferredoxin gene, a unique single guide RNA (sgRNA) (ATATCCATTAAGAATATGTT) under the control of the constitutive P*_araE_* promoter of the C. acetobutylicum *araE* gene targeting *nfn*, and a homologous recombination cassette to allow the precise in-frame deletion of *nfn*. Following vector assembly, the construct was transferred to wild-type *C. autoethanogenum* by conjugation using sExpress as the E. coli donor strain (45). Following two rounds of selection on thiamphenicol and d-cycloserine, to select for recipient strains harboring the CRISPR vector and counterselect the E. coli donor cells, respectively, a colony PCR screen was performed on the resultant colonies, amplifying from the genomic locus flanking the regions selected for homologous recombination. The screen revealed that the *nfn* knockout mutant was indeed present in the population, and the strain was subcultured for storage and preparation of genomic DNA. Sanger sequencing from a high-fidelity PCR product confirmed the precise in-frame deletion of *nfn*.

### Assessment of transposon vectors.

Transposon delivery vectors were transferred to *C. autoethanogenum* via conjugal transfer from sExpress and selected for on YTF agar plates supplemented with clarithromycin and d-cycloserine. Colonies were harvested from selection plates by flooding with phosphate-buffered saline (PBS), and the entire cell suspension was serially diluted and spread onto YTF agar plates supplemented with either clarithromycin and IPTG, thiamphenicol and IPTG, or clarithromycin to determine the transposition frequency and plasmid retention in the presence of IPTG.

### Transposon library creation.

The transposon-delivery vector pMTL-CW20 was transformed into the E. coli conjugative donor strain sExpress (45), which was used to transfer the plasmid into *C. autoethanogenum* C24. Twelve conjugations were performed simultaneously, producing a total of around 81,000 transconjugant colonies on YTF agar supplemented with d-cycloserine and clarithromycin. All transconjugants were pooled and plated onto YTF agar plates supplemented with IPTG, lactose, and thiamphenicol to select for transposon mutants and incubated for 72 h. Transposon mutant colonies were then harvested and inoculated to YTF broth supplemented with IPTG and thiamphenicol. The rich medium sequencing samples were taken from this liquid phase, which was used to inoculate PETC pyruvate medium. The PETC pyruvate culture was allowed to reach stationary phase before being used as inoculum for the bioreactor, where CO served as the carbon and energy source; a DNA sample was taken from PETC pyruvate at the point of bioreactor inoculation. Samples were taken from the bioreactor to check for the presence of pyruvate, monitor the OD, and serve as DNA samples for identification of insertion sites.

### Bioinformatics and metabolic modeling.

Experimentally confirmed essential genes were compared against metabolic modeling-derived essentiality. Lists of essential genes were generated using the BioTraDIS toolkit approach as previous described (47). A genome-scale model (GSM) of CO-fed *C. autoethanogenum* was handled using the COBRA Toolbox in MATLAB R2016b to predict gene essentiality (34, 48). Briefly, the wild-type model was subjected to flux balance analysis (FBA) by selecting the maximization of the biomass yield as the objective function (35). A gene is deemed essential when knocking it out made the biomass reaction carry no flux (49). Finally, Matthew’s correlation coefficient was used as a metric to assess the quality of the GSM predictions, where 1 is a perfect correlation between experimental and predicted gene essentiality, 0 is no correlation, and −1 is a perfect anticorrelation (37). The model and the scripts are available in GitHub (https://github.com/SBRCNottingham/C_auto_essentiality).

### Sequencing and bioinformatics.

Sequencing library preparation was performed as an amplicon library using a splinkerette adapter (47). Genomic DNA was fragmented to an average of 400 bp using a Covaris sonicator followed by bead purification using NEB sample preparation beads at a ratio of 1.5× beads to sample. Fragmented DNA was end repaired and A-tailed using the NEB Ultra II library preparation kit. Splinkerette adapters were ligated onto the end of A-tailed fragments with reagents from the Ultra II library preparation kit. A 1× bead purification was performed before an I-SceI digest step to cleave plasmid DNA between the library primer and P7 primer. Another 1× bead purification was performed before PCR amplification of the transposon junctions using KAPA HiFi polymerase. An initial denaturing step of 95°C for 2 min was followed by 20 rounds of 95°C for 20 s, 61°C for 30 s, and then 72°C for 30 s, before a final extension of 2 min at 72°C was performed.

PCR products with a size range of 250 to 500 bp were gel extracted from a low-melt agarose gel using the NEB monarch gel extraction kit. Gel-extracted products were analyzed on an Agilent bioanalyzer using a DNA 1000 chip and quantified via Qubit and quantitative PCR (qPCR). Two separate runs were performed on an Illumina MiSeq.

Raw sequences were trimmed before filtering for reads that contain the expected transposon tag. The transposon tag was removed from reads, which could then be mapped to the *C. autoethanogenum* genome to identify insertion sites. The BioTraDIS analysis pipeline was used for these steps and for subsequent analysis (47). Reads in the final 10% of the gene were omitted from the analysis. Reads that mapped to multiple locations on the genome were randomly mapped between those locations.

### Data availability.

Raw sequencing data have been deposited in NCBI under accession no. SRR16990784 to SRR16990788 and SRR17285607 to SRR17285609.
