# Supplementary material for: Required Gene Set for Autotrophic Growth of Clostridium autoethanogenum
Source: Appl Environ Microbiol. 2022 Mar 14;88(7):e02479-21. doi: 10.1128/aem.02479-21 (PMC9004356; doi:10.1128/aem.02479-21)
Supplement: Supplemental file 3 — Tables S3 to S13 and descriptions of Tables S1 and S2. Download aem.02479-21-s0001.pdf, PDF file, 0.3 MB [file aem.02479-21-s0001.pdf]

SUPPLEMENTARY INFORMATION

Application of transposon-insertion sequencing to determine gene essentiality in the acetogen *Clostridium autoethanogenum*

Craig Woods[1], Christopher M. Humphreys[1], Claudio Tomi-Andrino[1,2,3], Anne M. Henstra[1], Michael Köpke[4], Sean D. Simpson[4], Klaus Winzer[1], Nigel P. Minton[1,5]

[1] Clostridia Research Group, BBSRC/EPSRC Synthetic Biology Research Centre (SBRC), Biodiscovery Institute, School of Life Sciences, The University of Nottingham, Nottingham, NG7 2RD, UK

[2] Centre for Analytical Bioscience, Advanced Materials and Healthcare Technologies Division, School of Pharmacy, The University of Nottingham, Nottingham, NG7 2RD, UK

[3] BBSRC/EPSRC Synthetic Biology Research Centre (SBRC), School of Mathematical Sciences, The University of Nottingham, Nottingham, NG7 2RD, UK

[4] LanzaTech Inc., 8045 Lamon Avenue, Suite 400, Skokie, IL, USA

[5] NIHR Nottingham Biomedical Research Centre, Nottingham University Hospitals NHS Trust and the University of Nottingham, Nottingham, NG7 2RD, UK.

Correspondence and requests for materials should be addressed to Professor Nigel P Minton (email: nigel.minton@nottingham.ac.uk)

EXPERIMENTAL

Table S1: Essentiality status for each gene in the *C. autoethanogenum* genome. – see attached Excel File

Transposon libraries were tracked while passaged through rich medium, minimal medium with pyruvate as the carbon source, and finally minimal medium with CO as the carbon source. For each of these conditions a call is made on the essentiality status on each gene in *C. autoethanogenum*. If a gene was predicted to be essential on a previous growth condition then no assertion is made on the subsequent condition, the hyphen should be interpreted to mean an absence of a call being made on the essentiality status.

Table S2: Comparison of essentiality status calls from flux-balance analysis and transposon sequencing data.

A comparison of the essentiality status for the 534 genes considered in a genome scale model [1] of *C. autoethanogenum* as determined by flux-balance analysis and by the transposon sequencing experimental data.

ANALYTICS

Table S3: Bioreactor run data

The pyruvate concentration of the fermentation medium was measured via HPLC in order to accurately determine when autotrophic growth was occuring. Pyruvate was added to the initial fermentation broth in order to avoid unwanted bottleneck effects after inoculation of the transposon library.

Date (DD/MM/YYYY)	Elapsed Time	Pyruvate Concentration (mM)	Optical Density
29/11/2017 (Inoculation)	0	-	-
30/11/2017	24	12.56	-
02/12/2017	48	0.07	-
03/12/2017	72	0.05	0.218
04/12/2017	96	0.03	0.345
05/12/2017	120	0.04	0.403
06/12/2017	144	0.05	0.418
07/12/2017	168	0.06	0.423
08/12/2017	192	0.06	0.487
09/12/2017	216	0.06	0.579
10/12/2017	240	0.05	0.725
11/12/2017	264	0.04	0.928
12/12/2017	288	0.08	1.252
13/12/2017	312	0.06	1.559
14/12/2017	336	0.03	1.687
15/12/2017	360	0.03	2.358
16/12/2017	384	0.02	

MEDIA

Table S4: YTF Medium

Components from the table were added to a stirred vessel containing the desired volume double-distilled H_2O in the order that they appear in the table after which the pH was set to 5.8 using concentrated HCl.

Component	Quantity per litre
Yeast Extract	10 g
Tryptone	16 g
Fructose	10 g
NaCl	0.2 g
Acid 1000x trace element solution	1 ml
Basic 1000x trace element solution	1 ml
Vitamin 1000x stock solution	1 ml

Table S5: YTF Media Acid 1000x Trace element solution.

Components from the table were added to a stirred vessel containing the desired volume double-distilled H_2O in the order that they appear in the table.

Component	Concentration
HCl	50 mM
H_3BO_3	100 mg/L
$MnCl_2.4H_2O$	230 mg/L
$FeCl_2.4H_2O$	78 mg/L
$CoCl_2.6H_2O$	103 mg/L
$NiCl_2.6H_2O$	602 mg/L
$ZnCl_2$	78 mg/L
$CuSO_4.5H_2O$	50 mg/L
$AlK(SO_4)_2.12H_2O$	50 mg/L

Table S6: YTF Media 1000x basic trace element solution.

Components from the table were added to a stirred vessel containing the desired volume double-distilled H_2O in the order that they appear in the table.

Component	Quantity
NaOH	10 mM
Na_2SeO_3	58 mg/L
Na_2WO_4	53 mg/L
$Na_2MbO_4.2H_2O$	52 mg/L

Table S7: YTF Media Vitamin 1000x stock solution.

Components from the table were added to a stirred vessel containing the desired volume double-distilled H_2O in the order that they appear in the table.

Component	Concentration mg/L
p-aminobenzoate	114
Riboflavin	104
Thiamine	200
Nicotinate	206
Pyridoxin	510
Pantothenate (calcium)	104
Cyanocobalamin	78
d-biotin	22
Folate	48
Lipoate/thioctic acid	50

Table S8: PETC Medium.

Components from the table were added to a stirred vessel containing the desired volume of double-distilled H_2O in the order that they appear in the table after which the pH of the solution was set to pH 5.8 using concentrated NaOH.

Component	Concentration (g/L)
NH_4Cl	1.00 g/L
KCl	0.10 g/L
$MgSO_4.7H_2O$	0.20 g/L
KH_2PO_4	0.20 g/L
$CaCl_2$	0.02 g/L
Nitrilotriacetic Acid	0.05 g/L
$Fe(SO_4)_2(NH_4)_2$.6H2O	0.05 g/L
CH_3COONa	0.25 g/L
MES buffer	20.00 g/L
Trace metals stock (100x)	1% (v/v)
Wolfes vitamins stock (100x)	1% (v/v)
Resazurin stock (2 gL^{-1}) (2000x)	0.05% (v/v)

Table S9: PETC Medium Trace metal solution (100X)

The desired end volume of double-distilled H_2O was added to a stirred vessel before being set to pH 6 with concentrated KOH. Components were then added in the order that they appear in the table.

Trace metal solution (100X)	Grams per litre
Nitrilotriacetic Acid	2.00
MnSO4.H_2O	1.00
Fe $(SO_4)_2$(NH4)$_2$.6H_2O	0.80
$CoCl_2.6H_2O$	0.20
$ZnSO_4.7H_2O$	0.0002
$CuCl_2.2H_2O$	0.02
$NaMoO_4.2H_2O$	0.02
Na_2SeO_3	0.02
$NiCl_2.6H_2O$	0.02
$Na_2WO_4.2H_2O$	0.02

Table S10: PETC Medium Wolfe's vitamin solution (100x)

Components from the table were added in order to a stirred vessel containing double-distilled H_2O

Wolfe's vitamin solution (100x)	Grams per litre
Biotin	0.004
Folic acid	0.004
Pyridoxine hydrochloride	0.002
Thiamine.HCl	0.010
Riboflavin	0.010
Nicotinic acid	0.010
Calcium D-(+)-pantothenate	0.010
Vitamin B12	0.0002
p-Aminobenzoic acid	0.010
Thioctic acid	0.010

Table S11: Fermentation Medium.

Components were added to a stirred vessel containing the desired volume of double-distilled H_2O in the order that they appear in the table.

Media component	Amount per 1.0 L
$MgCl_2.6H_20$	0.5 g
$CaCl_2.2H_20$	0.37 g
KCl	0.15 g
NaCl	0.12 g
85% H_3P0_4	0.38ml
NH_4Cl	1 g
Metal mix 1	1 ml
Metal mix 2	0.2 ml
Tungsten solution	0.2 ml
Rezasurin (2 g/L)	0.5 ml
B-vitamin solution	10 ml

Table S12: Fermentation Medium Metal Mix 1 solution

Components from the table were added to a stirred vessel containing double-distilled H_2O

Component of stock	Amount per 1 L
$FeCl_2.4H_2O$	19.35 g
$NiCl_2.6H_2O$	1.19 g
$ZnCl_2$	0.69 g

Table S13: Fermentation Medium Metal Mix 2 solution

Components from the table were added to a stirred vessel containing double-distilled H_2O

Component of stock	Amount per 1 L
$CoCl_2$	2.38 g
HBO_4	0.62 g
$MnCl_2.4H_2O$	1.98 g
$NaMoO_4.2H_2O$	2.42 g
Na_2SeO_3	1.73 g

Reference

1. Norman R, Millat T, Schatschneider S, Henstra AM, Breitkopf R, Pander B, Annan FJ, Piatek P, Hartman HB, Poolman MG, Fell DA, Winzer K, Minton NP, Hodgman C. Genome-scale model of *C. autoethanogenum* reveals optimal bioprocess conditions for high-value chemical production from carbon monoxide. *Eng. Biol.* **3**, 32–40 (2019).
